# BL-038, a Benzofuran Derivative, Induces Cell Apoptosis in Human Chondrosarcoma Cells through Reactive Oxygen Species/Mitochondrial Dysfunction and the Caspases Dependent Pathway

**DOI:** 10.3390/ijms17091491

**Published:** 2016-09-07

**Authors:** Ju-Fang Liu, Chien-Yu Chen, Hsien-Te Chen, Chih-Shiang Chang, Chih-Hsin Tang

**Affiliations:** 1 Central Laboratory, Shin-Kong Wu Ho-Su Memorial Hospital, Taipei 111, Taiwan; anti0822@hotmail.com; 2 School of Pharmacy, College of Pharmacy, China Medical University, Taichung 404, Taiwan; qaz90006@yahoo.com.tw; 3 Department of Orthopedic Surgery, China Medical University Hospital, Taichung 404, Taiwan; bonekid1@gmail.com; 4 School of Chinese Medicine, China Medical University, Taichung 404, Taiwan; 5 Graduate Institute of Basic Medical Science, China Medical University, Taichung 404, Taiwan; 6 Department of Pharmacology, School of Medicine, China Medical University, Taichung 404, Taiwan; 7 Department of Biotechnology, College of Health Science, Asia University, Taichung 413, Taiwan

**Keywords:** chondrosarcoma, benzofuran derivative, ROS, apoptosis

## Abstract

Chondrosarcoma is a highly malignant cartilage-forming bone tumor that has the capacity to invade locally and cause distant metastasis. Moreover, chondrosarcoma is intrinsically resistant to conventional chemotherapy or radiotherapy. The novel benzofuran derivative, BL-038 (2-amino-3-(2,6-dichlorophenyl)-6-(4-methoxyphenyl)benzofuran-4-yl acetate), has been evaluated for its anticancer effects in human chondrosarcoma cells. BL-038 caused cell apoptosis in two human chondrosarcoma cell lines, JJ012 and SW1353, but not in primary chondrocytes. Treatment of chondrosarcoma with BL-038 also induced reactive oxygen species (ROS) production. Furthermore, BL-038 decreased mitochondrial membrane potential (MMP) and changed mitochondrial-related apoptosis, by downregulating the anti-apoptotic activity members (Bcl-2, Bcl-x_L_) and upregulating pro-apoptotic members (Bax, Bak) of the B-cell lymphoma 2 (Bcl-2) family of proteins, key regulators of the apoptotic machinery in cells. These results demonstrate that in human chondrosarcoma cells, the apoptotic and cytotoxic effects of BL-038 are mediated by the intrinsic mitochondria-mediated apoptotic pathway, which in turn causes the release of cytochrome c, the activation of caspase-9 and caspase-3, and the cleavage of poly (ADP-ribose) polymerase (PARP), to elicit apoptosis response. Our results show that the benzofuran derivative BL-038 induces apoptosis in chondrosarcoma cells.

## 1. Introduction

Chondrosarcomas are the third most common primary malignancy of bone after myeloma and chondrosarcoma; comprising a heterogeneous group of neoplasms that are characterized by cartilage matrix production from the tumor cells [1]. Patients with malignant chondrosarcomas frequently develop pulmonary metastasis, which represents approximately 10% of all primary bone tumors and is associated with usually poor prognosis [2]. As chondrosarcomas responds poorly to chemotherapy and radiotherapy, wide surgical excision remains the treatment of choice [2]. In the absence of an effective therapy, novel and adequate therapeutic approaches are needed [3].

Reactive oxygen species (ROS) regulate multiple cellular events such as inflammation [4], cell cycle progression [5], apoptosis [6], migration, and invasion [7]. Excessive ROS production can induce apoptosis in various cancer cell lines [8,9]. Intrinsic mitochondria apoptosis occurs in response to death stimuli, including activation of chemotherapeutic agents [10], serum starvation [11], ultraviolet radiation [12] and ROS [13]. Production of high levels of ROS is known to cause mitochondrial DNA damage, mitochondrial membrane permeabilization, and the release of cytochrome *c* from mitochondria, triggering caspase-dependent or caspase-independent cytosolic signaling events [14,15].

Benzofuran is considered to be an important class of heterocyclic compound, possessing a variety of biological and pharmacological properties that include anti-inflammatory, antioxidant, antimicrobial, antifungal, antihyperglycemic, analgesic, antiparasitic, and antitumor activities [16,17,18,19]. Some benzofuran derivatives have shown potential as therapeutic agents for human cancers. For instance, Li et al. [20] have provided evidence suggesting that synthesized 3-acyl-5-hydroxybenzofuran derivatives exhibit anti-proliferative effects against human breast cancer MCF-7 cells. However, the role of benzofuran derivatives in chondrosarcoma cells remains largely undefined. There are well known natural products that are related benzofuran scaffold. In this study, we synthesized 39 novel benzofuran derivatives and subjected to screen the activity against human chondrosarcoma cells. Finally, 2-amino-3-(2,6-dichlorophenyl)-6-(4-methoxyphenyl)benzofuran-4-yl acetate (BL-038) possessed a potent inhibitory activity. Our findings indicate that BL-038 decreases cell survival and tumor growth in vitro.

## 2. Results

### 2.1. BL-038 Inhibits the Growth of Human Chondrosarcoma Cells

**Figure 1:**
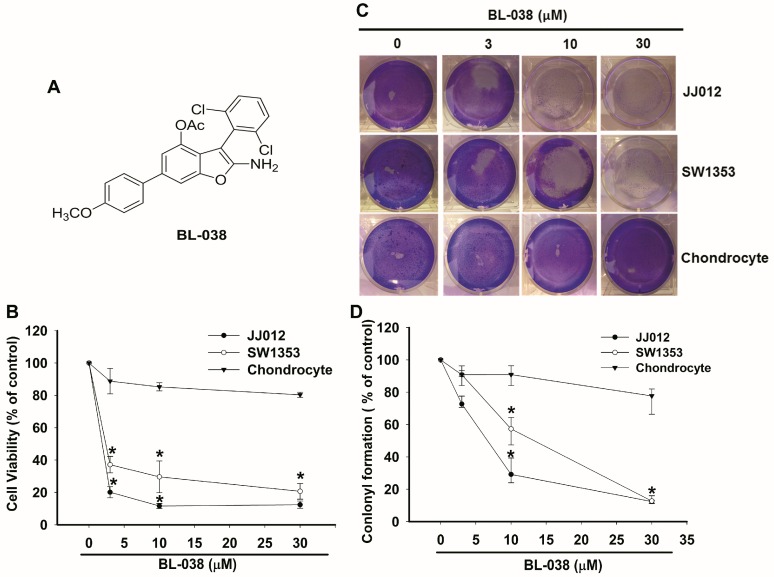
2-Amino-3-(2,6-dichlorophenyl)-6-(4-methoxyphenyl)benzofuran-4-yl acetate (BL-038) decreases cell viability in chondrosarcoma cells: (**A**) The structure of BL-038; (**B**) JJ012 and SW1353 chondrosarcoma cells, as well as chondrocytes, were treated with indicated concentrations of BL-038 for 48 h, and cell viability was assessed by 3-(4,5-Dimethylthiazol-2-yl)-2,5-diphenyltetrazolium bromide (MTT) assay; and (**C**,**D**) Cells were incubated with BL-038 for 7 days. Colony formation assay on the cells was performed and stained using crystal violet and photographed. The quantitative data are shown in (**D**). Results are expressed as the mean ± SEM (the standard error of the mean). * *p* < 0.05 compared with controls.

The chemical structure, 2-amino-3-(2,6-dichlorophenyl)-6-(4-methoxyphenyl)benzofuran-4-yl acetate (BL-038), was synthesized at the Graduate Institute of Pharmaceutical Chemistry, China Medical University and is represented in Figure 1A. The 3-(4,5-Dimethylthiazol-2-yl)-2,5-diphenyltetrazolium bromide (MTT) assay was used to examine the cell death effects of BL-038 on human chondrosarcoma cells. Human chondrosarcoma cells (JJ012 and SW1353) were treated with 3, 10 and 30 µM BL-038 for 48 h; BL-038 induced cell death in a concentration-dependent manner (Figure 1B). The half maximal inhibitory concentration (IC_50_) values of BL-038 were 1.8 and 2.2 µM for JJ012 and SW1353 cells, respectively. BL-038 did not affect the viability of normal primary chondrocytes. BL-038 anticancer activities were further assessed with an in vitro clonogenic cell survival assay, which correlated very well with previous in vivo assays of tumorigenicity in nude mice [21]. JJ012 and SW1353 cells pretreated with 3, 10 and 30 µM BL-038 exhibited significantly lower clongenic survival fractions than cells treated with vehicle, in which the addition of BL-038 led to a dose-dependent inhibition in clonogenicity (Figure 1C,D).

### 2.2. BL-038 Induces Apoptosis and Cell Migration in Human Chondrosarcoma Cells

**Figure 2:**
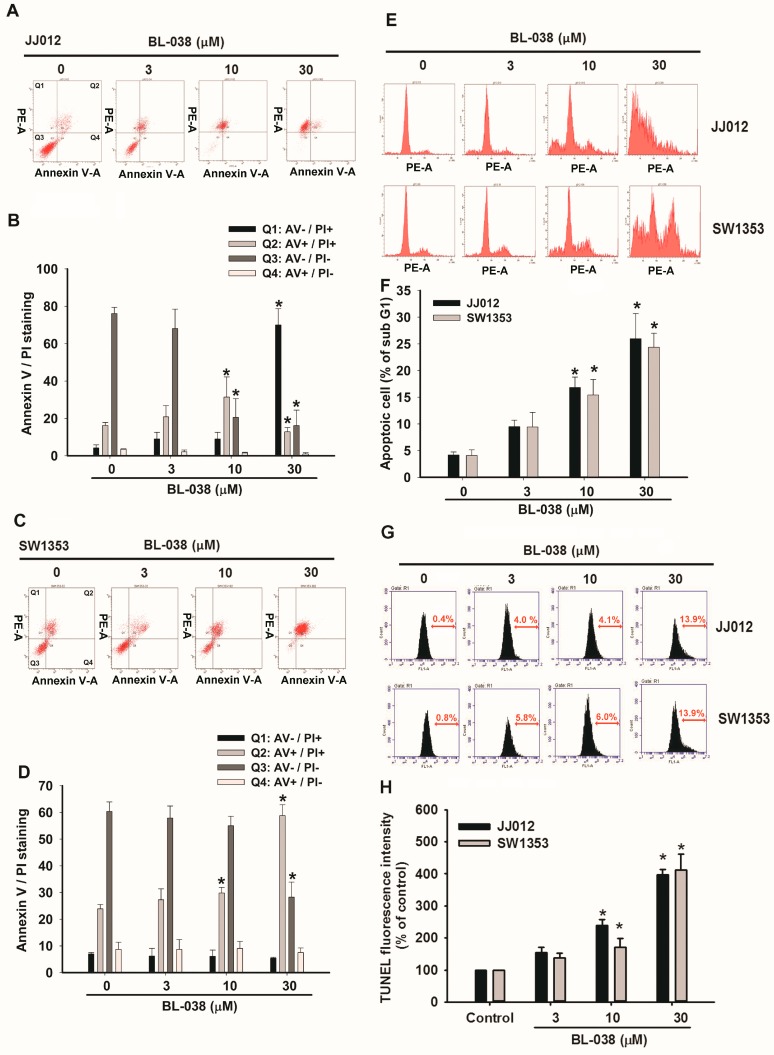
BL-038 induces cell apoptosis in chondrosarcoma cells. (**A**–**D**) The JJ012 and SW1353 chondrosarcoma cells were incubated with indicated conditions of BL-038 for 24 h, the cells were stained by annexin V/PI and percentage of apoptotic cells were analyzed by flow cytometric analysis; (**E**,**F**) cells were treated as described in (**A**), the cells were stained by propidium iodide (PI) and the apoptotic cells were assessed by flow cytometric analysis; (**G**,**H**) cells were treated with vehicle or BL-038 for 24 h. The terminal deoxynucleotidyl transferase dUTP nick end labeling (TUNEL) positive cells were examined by flow cytometry. Results are expressed as the mean ± SEM. * *p* < 0.05 compared with controls.

We next investigated whether reduced clonogenic survival in the presence of BL-038 was associated with increased apoptosis. This assay is based on evaluating apoptotic cells by detecting the phosphatidylserines (PS) externalization, a hallmark of the early phase of apoptosis. Annexin V-FITC (fluorescein isothiocyanate) is a fluorescent probe that binds to phosphatidylserine. Figure 2A–D shows that annexin V-FITC/PI double-positive cells increased at 24 h after treatment with BL-038 at 3, 10 and 30 µM in JJ012 and SW1353 cells. Next, we investigated the mechanism by which BL-038 induced cell apoptosis in JJ012 and SW1353 cells. We found that BL-038 markedly increased the sub-G1 cell population (Figure 2E,F). Treatment of JJ012 cells with BL-038 at 3, 10 and 30 μM for 24 h resulted in the accumulation of cells in the sub-G1 phase from 3.8% in the untreated control cells to 9.7%, 18.8% and 27.2%, respectively. When we applied the terminal deoxynucleotidyl transferase-mediated deoxyuridine triphosphate nick end labeling (TUNEL) assay, we found that BL-038 induced a significant increase in cells with clear features of apoptosis (Figure 2G,H). These results indicate that the accumulation of the apoptotic population of chondrosarcoma by BL-038 may be responsible for the inhibition of cell growth.

### 2.3. Reactive Oxygen Species (ROS) Are Involved in BL-038-Induced Apoptosis in Human Chondrosarcoma Cells

**Figure 3:**
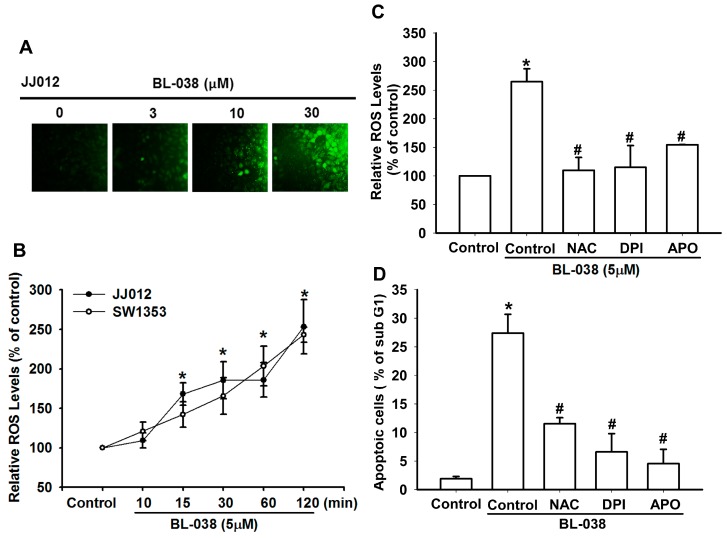
BL-038 induces ROS production in chondrosarcoma cells. (**A**,**B**) Cells were treated with indicated concentrations of BL-038, the ROS generation was assessed by CM-H2DCFDA staining kit staining, and the stained cells were performed with flow cytometric analysis; (**C**,**D**) cells were pretreated with *N*-acetylcysteine (NAC), Diphenylene iodonium (DPI) and Apocynin (APO) for 30 min, then the cells were incubated with BL-038 (5 µM). The percentage of ROS production and apoptotic cells were assessed by CM-H2DCFDA staining kit and PI staining. Results are expressed as the mean ± SEM. * *p* < 0.05 compared with controls. # *p* < 0.05 compared with BL-038 treated groups.

ROS generation plays an important role in apoptosis [22]. We therefore investigated whether the accumulation of ROS is involved in BL-038-induced cell apoptosis. Cells were exposed to BL-038 at 3, 10 and 30 µM for 30 min and analyzed for the production of ROS by fluorescence microscopy following staining with CM-H2DCFDA (Figure 3A). FACS analysis indicated that treatment of chondrosarcoma cells with BL-038 (5 µM) for 10–120 min induces the accumulation of ROS (Figure 3B). *N*-acetylcysteine (NAC, NADPH oxidase inhibitor), diphenyleneiodonium chloride (DPI, non-specific flavoprotein inhibitor), and apocynin (APO, NOX-like enzymes inhibitor) reduced BL-038-induced ROS production and cell apoptosis (Figure 3C,D). These results demonstrate that BL-038 induces apoptosis in chondrosarcoma cells via ROS production.

### 2.4. Involvement of Mitochondrial Dysfunction in BL-038-Induced Human Chondrosarcoma Cell Apoptosis

**Figure 4:**
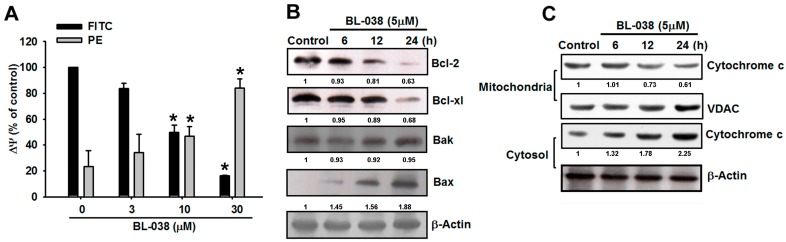
BL-038 induces mitochondrial dysfunction in human chondrosarcoma cells: (**A**) Cells were incubated with BL-038 for 24 h, mitochondrial membrane potential (MMP) was satiated with JC-1 and examined by flow cytometry; (**B**) JJ012 cells were incubated with BL-038 (5 μM) for different time intervals, the Bax, Bak, Bcl-2, and Bcl-x_L_ expressions were examined by Western blot analysis; and (**C**) JJ012 cells were incubated with BL-038 (5 μM) for different time intervals, the levels of cytochrome *c* in mitochondria and cytosol were examined by Western blot analysis. Results are expressed as the mean ± SEM. * *p* < 0.05 compared with controls.

After confirming the apoptotic effect of BL-038 on chondrosarcoma cells, we then explored whether BL-038-induced cell apoptosis is mediated through mitochondrial dysfunction. Mitochondrial membrane protein (MMP) was determined using the mitochondria-sensitive fluorescent dye, JC-1, by flow cytometry. The cyanine dye JC-1 is a cationic dye that accumulates in energized mitochondria. In healthy non-apoptotic cells, JC-1 accumulated as aggregates in the mitochondrial membranes, resulting in red fluorescence (PE). Apoptotic cells showed primarily green fluorescence (FITC). Treatment of JJ012 cells with BL-038 induced marked changes in MMP, as demonstrated by the disappearance of red fluorescence or the increase of green fluorescence (Figure 4A). The mitochondrial pathway of apoptosis involves signaling by mitochondrial-related apoptotic proteins, including B-cell lymphoma 2 (Bcl-2), Bcl-x_L_, Bak, Bax and the mitochondrial release of cytochrome *c* into the cytosol. Treatment of JJ012 cells with BL-038 (5 µM) increased Bax and Bak levels (Figure 4B) and reduced Bcl-x_L_ and Bcl-2 expression (Figure 4B), which increased the ratio of pro-apoptotic/anti-apoptotic Bcl-2. Furthermore, BL-038 (5 µM) enhanced levels of cytosolic cytochrome *c* and reduced mitochondrial cytochrome *c* expression (Figure 4C). These data suggest that BL-038 induced cell apoptosis through mitochondrial dysfunction in human chondrosarcoma cells.

### 2.5. BL-038 Induces the Activation of Caspases in Human Chondrosarcoma Cells

**Figure 5:**
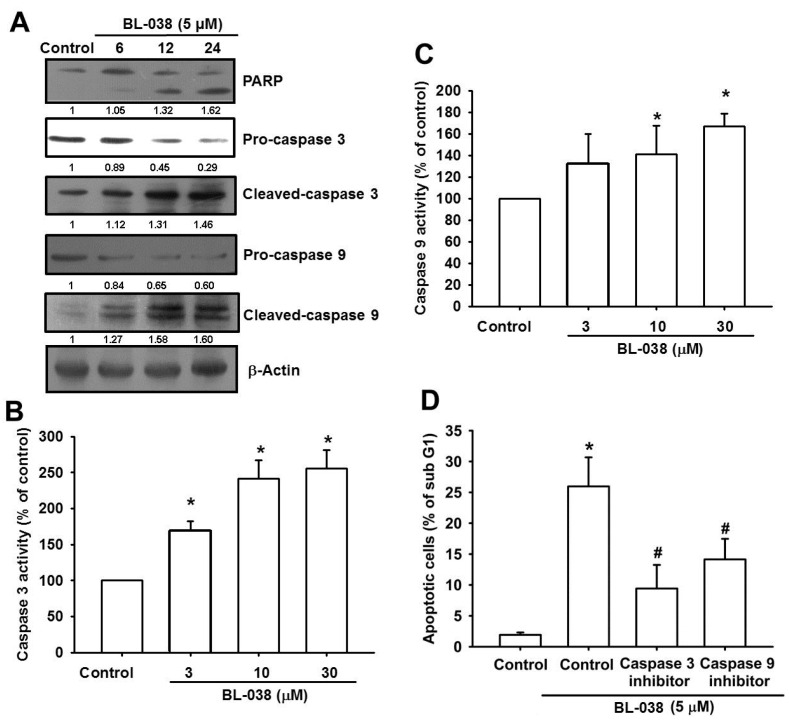
Effects of BL-038 on caspase activation: (**A**) JJ012 cells were treated with BL-038 (5 μM) for the indicated times. PARP, procaspase 3, cleaved-caspase 3, procaspase 9, cleaved-caspase 9 and β-actin levels were analyzed by Western blot; (**B**,**C**) JJ012 cells were treated with BL-038 for 24 h, and the caspases activities were examined; and (**D**) JJ012 cells were pre-treated with the indicated inhibitors for 30 min, then incubation with BL-038 (5 μM) for 24 h. The percentage of apoptotic cells were then analyzed by flow cytometric analysis of PI-stained cells. Results are expressed as the mean ± SEM. * *p* < 0.05 compared with controls. # *p* < 0.05 compared with BL-038 treated groups.

Caspases are a family of cysteine protease enzymes that play an essential role in programmed cell death [23]. The proteolytic cascade of caspases mediates cell apoptosis. We therefore examined the involvement of caspases in BL-038-induced apoptosis. In cells treated with BL-038 (5 µM), the levels of cleaved-PARP, caspase-3 and caspase-9 significantly increased (Figure 5A). Upstream caspase-3 and caspase-9 activities increased significantly, as shown by the observation that treatment with BL-038 (5 µM) increased caspase-3 and caspase-9 activity in chondrosarcoma cells (Figure 5B,C). Furthermore, we also found that pretreatment with the specific caspase-3 inhibitor (z-DEVD-FMK) or the specific caspase-9 inhibitor (z-LEHD-FMK) prevented cell apoptosis in cells treated with BL-038 (5 µM) (Figure 5D). These results demonstrate that BL-038 induces apoptosis in chondrosarcoma cells via caspase-dependent pathways.

### 2.6. BL-038 Reduces Cell Migration and Angiogenesis by Decreasing the Expression of Matrix Metalloproteinase-9 and Vascular Endothelial Growth Factor in Human Chondrosarcoma Cells

**Figure 6:**
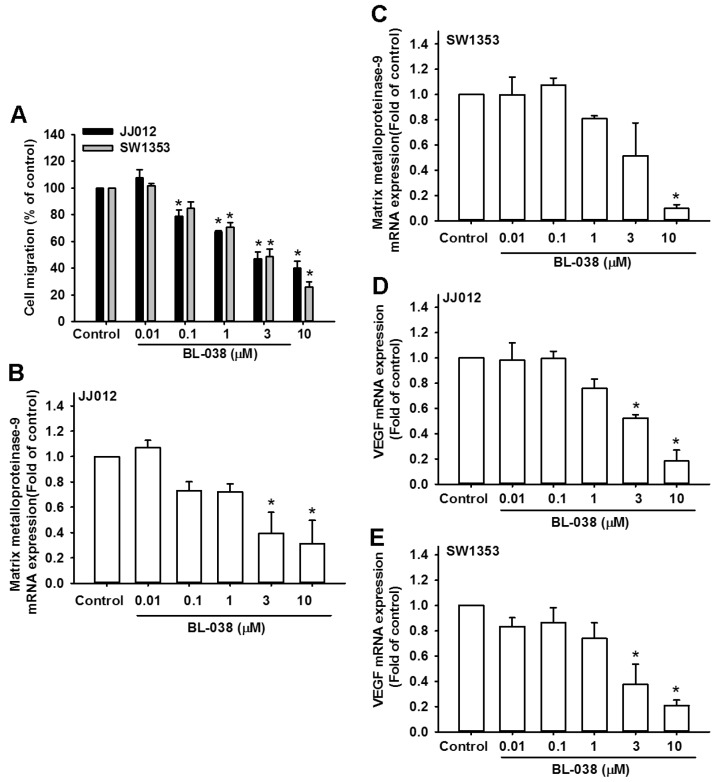
BL-038 inhibits cell migration and angiogenesis of human chondrosarcoma cells by modulating the expression of matrix metalloproteinase-9 and VEGF: (**A**) Chondrosarcoma cells (JJ012 and SW1353) were incubated with control solution or various concentrations of BL-038 for 24 h, then migration and invasion were measured in vitro using Transwell assays; and (**B**–**E**) Chondrosarcoma cells (JJ012 and SW1353) were incubated with control solution or various concentrations of BL-038 for 24 h; the expression of matrix metalloproteinase-9 and VEGF mRNAs was examined by qPCR. The data are expressed as the mean ± SEM. * *p* < 0.05 compared with controls.

Previous reports have indicated that chondrosarcoma cells have high metastatic potential [24,25]. We therefore examined the effects of BL-038 on the migration phenotype of chondrosarcoma cells. We found that low doses of BL-038 inhibited cell migration (Figure 6A). Next, we investigated which migratory component was inhibited by BL-038. Matrix metalloproteinase-9 and vascular endothelial growth factor (VEGF) have been shown to be pivotal proteins that participate in tumor cell migration, invasion, and metastasis [26,27], and both may play a crucial role in the metastasis of chondrosarcoma [28,29]. As we expected, BL-038 treatment decreased the expression of matrix metalloproteinase-9 and VEGF mRNA in chondrosarcoma cell lines (Figure 6B–E). These results reveal that BL-038 may regulate migration and angiogenesis of chondrosarcoma cells through matrix metalloproteinase-9 and VEGF.

## 3. Discussion

Benzofuran is a heterocyclic ring compound that has emerged as a powerful scaffold for multiple biological activities. Some studies have reported that benzofuran derivatives have potential antitumor activity against many types of cancer, such as tongue squamous cell carcinoma [30], bladder cancer [31], and breast cancer [32]. We have shown that BL-038, a novel synthetic small molecular benzofuran derivative compound, is effective in inducing cell apoptosis in the human chondrosarcoma cell line through ROS, mitochondrial dysfunction and caspase-dependent pathways. Moreover, we determined the exact mechanism of BL-038-induced apoptosis.

Chondrosarcoma is a bone malignancy that accounts for 10%–20% of primary bone tumors [33]. Surgical treatment of chondrosarcoma is effective for chondrosarcoma, achieving better clinical outcomes than either conventional radiotherapy or chemotherapy. Grade I tumors have a lower incidence of metastases and better prognosis as compared with high-grade chondrosarcoma [34]. The prognosis of high-grade chondrosarcoma is poor even after adequate surgery. Thus, agents that inhibit cell growth and induce cell death may be useful in the treatment of chondrosarcoma. In this study, we showed that growth of human chondrosarcoma cells (JJ012 and SW1353) was inhibited by BL-038 at concentrations ranging from 3 to 30 μM for 48 h. BL-038 significantly decreased the proliferation of JJ012 and SW1353 cells in a concentration-dependent manner (Figure 1). Here we found that BL-038 inhibited cell apoptosis in two different human chondrosarcoma cell lines but not normal chondrocytes. These results indicate that BL-038 may be more efficient at inducing cell apoptosis in chondrosarcoma. It remains to be determined as to whether normal chondrocytes express higher levels of protective and anti-apoptotic molecules as compared with levels found in chondrosarcoma cells. Furthermore, to understand the association between BL-038 and apoptosis, we examined the levels of various apoptotic markers in JJ012 and SW1353 cells after exposure to 3, 10 and 30 μM of BL-038. We found that apoptosis was induced by BL-038 in a concentration-dependent manner, as shown by annexin V/propidium iodide staining, sub-G1 populations in the cell cycle assay and TUNEL staining (Figure 2).

The mechanism of apoptosis can be initiated by two major apoptotic pathways, the intrinsic pathway (mitochondrial-mediated) and the extrinsic pathway (death receptor-mediated) [23,35]. The extrinsic or death receptor pathway activates the Fas ligand (FasL) receptor and recruits Fas-Associated protein with Death Domain (FADD) and caspase-8 [36]. The intrinsic mitochondrial pathway is activated by multiple stimuli that converge at the mitochondrion and induce MMP and subsequently result in the release of pro-apoptotic mitochondrial proteins into the cytosol [37]. The pro-apoptotic proteins include proteins that can activate the caspase-dependent pathway [37]. This group includes cytochrome *c* (cyt *c*). Release of cyt *c* activates Apaf-1, which subsequently activates a downstream caspase program [38]. Bcl-2 prevents the release of cyt *c* into the cytoplasm and thus blocks cyt *c* from promoting Apaf-1-mediated caspase-9 activation, leading some to hypothesize that Bcl-2 and its homologues function to keep mitochondrial membranes intact [39].

Evidence indicates that the mitochondrial pathway is activated by a benzofuran derivative [40]. In 2009, Kim et al. provided evidence showing that silvestrol induced an apoptotic response, disrupted the mitochondrial trans-membrane potential and caused cytochrome *c* release into the cytoplasm in LNCaP cells [41]. Moreover, it has also been reported that benzbromarone (a benzofuran derivative, contains heterocyclic ring compound) causes mitochondrial dysfunction in HepG2 cells and primary human hepatocytes [42]. Here, we hypothesized that mitochondrial-initiated ROS activation mediates BL-038-induced cell death. Disruption of MMP results in release of cytochrome *c* from mitochondria into the cytoplasm. In our experiment, the loss of MMP and increased cytochrome *c* release (Figure 4) confirmed our hypothesis.

Oxidative stress is considered to be an important pathogenic mechanism [23]. Oxidative stress results from an imbalance between free radical generation and antioxidant defenses. Oxidative stresses refer to elevate the levels of intracellular ROS that cause damage to lipids, proteins and DNA and other macromolecules that can regulate initiation of apoptotic signaling [43,44]. Studies evaluating the role of benzofuran derivative can induce ROS generation [45,46]. ROS produced by chondrosarcoma cells after BL-038 treatment were observed whereas pretreatment with NAC, DPI, and APO for 30 min blocked the ROS production and cell apoptosis (Figure 3). These results demonstrated that ROS are crucial factor in the induction of apoptosis and act upstream signaling molecules to initiate cell apoptosis.

ROS induce cell apoptosis by regulating the proapoptotic Bcl-2 family proteins, such as Bcl-2-associated X protein (Bax) and Bcl-2-antagonistic/killer (Bak), resulting in increased mitochondrial membrane permeabilization and cytochrome *c* released into the cytosol [47]. We observed a decrease in expression of Bcl-2 and Bcl-x_L_, as well as an increase in expression of Bax and Bak, in chondrosarcoma cells after treatment BL-038 (Figure 4). We also measured the change of mitochondrial membrane potential (MMP) induced by BL-038 stimulation. BL-038 could induce depolarization of the inner mitochondrial membrane in a dose-dependent manner. Therefore, the mitochondrial dysfunction is involved in BL-038-mediated cell apoptosis in chondrosarcoma.

Cancer metastasis occurs in complex multiple steps and is the main cause of treatment failure. A fundamental step in the metastasis process is the proteolytic degradation of matrix-degrading proteases such as matrix metalloproteinases [48]. In particular, matrix metalloproteinase-9 overexpression has been associated with the progression and invasion of different types of tumors, including mammary tumors [49]. VEGF is a signal protein that regulates vasculogenesis and angiogenesis. The overexpression of VEGF may be an early step in the process of metastasis [50]. The expression and tumor-promoting functions of matrix metalloproteinase-9 and VEGF have been well studied [51]. The present study showed that BL-038 inhibited migration and angiogenesis of chondrosarcoma cells by decreasing the expression of matrix metalloproteinase-9 and VEGF. Our data indicate that BL-038 regulates the expression of matrix metalloproteinase-9 and VEGF. Future research is warranted, to clarify whether additional signaling cascades are modulated by BL-038.

Here, we provide a new opportunity by using benzofuran derivative BL-038 in chondrosarcoma therapy. BL-038 induced cell apoptosis through activating ROS, mitochondria dysfunction and finally caspase activation. In summary, our present study shows that BL-038 may be a potential anti-cancer drug in chondrosarcoma treatment.

## 4. Materials and Methods

### 4.1. Materials

2-Amino-3-(2,6-dichlorophenyl)-6-(4-methoxyphenyl)benzofuran-4-yl acetate (BL-038: Figure 1A) was synthesized at the Graduate Institute of Pharmaceutical Chemistry, China Medical University (Taichung, Taiwan). Horseradish peroxidase-conjugated anti-mouse and anti-rabbit IgG, and rabbit polyclonal antibodies specific for cytochrome *c*, Bcl-2, Bcl-x_L_, Bax, Bak, PARP, caspase 3, caspase 9 and β-actin were purchased from Santa Cruz Biotechnology (Santa Cruz, CA, USA). All other chemicals were obtained from Sigma-Aldrich (St. Louis, MO, USA).

### 4.2. Synthesized Method of 2-Amino-3-(2,6-dichlorophenyl)-6-(4-methoxyphenyl)benzofuran-4-yl Acetate (BL-038)

To a solution of 1-aryl-2-nitroethylene (1.0 equiv) and 4-methoxyphenyl cyclohexane-1,3-dione (1.5 equiv) in dry tetrahydrofuran (THF, 10 mL) was added Et_3_N (0.2 equiv) under argon. The mixture was stirred at room temperature for 6–12 h followed by the addition of Et_3_N (2.0 equiv), DMAP (0.2 equiv) and Ac_2_O (2.0 equiv). The resulting mixture was stirred at room temperature for 5–10 h. After completion as monitored by TLC, the resulting mixture was concentrated in vacuo. The residue was purified by flash chromatography on silica gel using *n*-hexane–dichloromethane as eluent to provide light-pink powder of BL-038. Yield 88%. mp 197.1–197.6 °C; ^1^H NMR (400 MHz, DMSO-*d*_6_) δ 7.57 (d, *J* = 8.8 Hz, 2 H, 2″, 6″-H), 7.54 (d, *J* = 8.1 Hz, 2 H, 3′, 5′-H), 7.46 (d, *J* = 1.1 Hz, 1 H, 5-H), 7.38–7.42 (m, 1 H, 4′-H), 7.02 (d, *J* = 1.1 Hz, 1 H, 7-H), 6.99 (d, *J* = 8.8 Hz, 2 H, 3″, 5″-H), 6.53 (brs, 2 H, NH2), 3.78 (s, 3 H, OCH3), 1.54 (s, 3 H, CH3); ^13^C NMR (125 MHz, DMSO-*d*_6_) δ 168.2, 158.4, 157.0, 150.7, 140.4, 137.5, 137.5, 132.4, 131.4, 130.1, 129.7, 127.8, 127.8, 127.4, 127.4, 122.4, 114.7, 114.3, 114.3, 104.8, 83.3, 55.1, 19.4; EIMS *m*/*z* 441.1 (M^+^). HPLC purity 99.6% (λ_max_ = 212 nm).

### 4.3. Cell Culture

Primary human articular chondrocytes were isolated in cell culture by collagenase treatment of tissue obtained during knee replacement surgeries of patients with osteoarthritis (OA), as we have described previously [52]. The cells were maintained in Dulbecco’s Modified Eagle Medium (DMEM) culture medium supplemented with HEPES (20 mM), fetal bovine serum (FBS; 10%), glutamine (2 mM), streptomycin (100 μg/mL) and penicillin (100 U/mL).

The human chondrosarcoma cell line JJ012 was provided by Dr. Sean P Scully (University of Miami School of Medicine, Miami, FL, USA) and the human chondrosarcoma cell line SW1353 was purchased from American Type Culture Collection (ATCC; Manassas, VA, USA). Cells were cultured in DMEM/α-MEM supplemented with FBS (10%), streptomycin (100 μg/mL) and penicillin (100 U/mL), then maintained at 37 °C in humidified air (95% air, 5% CO_2_).

### 4.4. MTT Assay

The 3-(4,5-dimethylthiazol-2-yl)-2,5-diphenyltetrazolium bromide (MTT) assay was used to assess cell viability. After undergoing treatment with BL-038 for 48 h, cultures were washed with PBS. Then MTT (0.5 mg/mL) was then added to each well and the mixture was incubated at 37 °C for 30 min. To analyze the spectroscopic properties of MTT formazan crystals, MTT was dissolved in an equal volume of DMSO and then absorbance of each well was determined at 550 nm using a microplate reader (Bio-Tek, Winooski, VT, USA).

### 4.5. Colony Formation Assay

Cells (1000 per well) were treated simultaneously for 3 h with BL-038 at various concentrations (3–30 µM) in a 6-well plate. Cells were allowed to grow for 7 days to form colonies before being stained with crystal violet (0.4 g/L). After washing with ddH_2_O for several times, acetic acid 33% (*v*/*v*) was added, and the absorbance was measured at 550 nm [53].

### 4.6. Annexin V/PI Staining

Cells (1 × 10^6^ per well) were treated with BL-038 for the indicated times, centrifuged at 1500 rpm for 5 min, washed twice with PBS, and resuspended in 0.5 mL binding buffer (10 mM HEPES pH 7.4, 140 mM NaCl, 2.5 mM CaCl_2_, 1 mM MgCl_2_, 5 mM KCl) containing 1 μg/mL propidium iodide (PI) and 0.025 μg/mL annexin V-FITC. Double-labeling was performed at room temperature for 10 min in the dark, and cells were analyzed by flow cytometry within 30 min, using an Epics Elite analyzer (Beckman-Coulter, Miami, FL, USA) [54].

### 4.7. Cell Cycle Analysis

Cells (1 × 10^6^ per well) were treated with BL-038 (3–30 µM) for the indicated times, centrifuged at 1500 rpm for 5 min, washed twice with PBS, resuspended and fixed overnight in 70% ethanol at −20 °C. Ethanol was then removed by centrifugation, and cellular DNA was stained with 100 μg/mL PI (in PBS containing 0.1% Triton-X-100, and 1 mM EDTA) in the presence of an equal volume of DNase-free RNase A (200 μg/mL) for 30 min in the dark. Cells were then analyzed immediately with a FACScan and Cellquest program (Becton Dickinson, San Jose, CA, USA). The extent of apoptosis was determined by measuring the DNA content of cells below sub G1 peak on DNA profiles [55].

### 4.8. TUNEL Staining

Detection of DNA-strand breaks during apoptosis was examined by TUNEL assay, according to the manufacturer’s instructions (Sigma, St. Louis, MO, USA). Cells were incubated with BL-038 for 24 h, fixed with 1% paraformaldehyde in PBS for 15 min at 4 °C. After washes with PBS, the pellet was resuspended in 1 mL 70% cold ethanol at −20 °C for 30 min to permeabilize cells. The cells were washed and incubated with 50 μL labeling solution containing fluorescein dUTP and terminal deoxynucleotidyl transferase (TdT) at 37 °C for 1 h. The stained cells were then analyzed by flow cytometry using a 488 nm argon-ion laser. Green fluorescence was detected using an emission filter of 530/40 nm.

### 4.9. Determination of ROS Generation

Levels of ROS generation were observed by the fluorogenic probe, dichlorodihydrofluorescein (CM-H2DCFDA; Thermo Fisher; Waltham, MA, USA). Cells were plated at a density of 2 × 10^5^ and were exposed to BL-038 for specified time intervals. The cells were stained with CM-H2DCFDA (5 μM) for 30 min at 37 °C, and the fluorescence intensity in cells was determined by flow cytometry and fluorescence microscopy.

### 4.10. Determination of Mitochondrial Membrane Potential

Cells (2 × 10^5^ per well) were plated in 6-well culture dishes, and treated with BL-038 (3–30 μM). After washing with PBS, cells were stained for 15 min in JC-1 (5 μg/mL) at 37 °C. The stained cells were analyzed using the Cellquest program.

### 4.11. Western Blot Analysis

Cells were plated in 6-well culture dishes, grown to confluence, and treated with BL-038. After incubation, cells were washed again with ice-cold PBS, scraped, pelleted and lysed in a radioimmunoprecipitation assay (RIPA) buffer (protease inhibitor cocktail and phosphatase inhibitor). After incubation for 1 h on ice, cell lysates were centrifuged at 14,000 rpm for 30 min at 4 °C. Lysate protein concentrations were determined by a BCA protein assay kit (Thermo Scientific, Hudson, NH, USA) and the lysates were adjusted with a lysis buffer. Proteins were resolved on a 10%–15% SDS-PAGE and transferred to Immobilon polyvinyldifluoride (PVDF) membranes. The blots were blocked with blocking buffer for 10 min at room temperature and then probed with rabbit anti-human antibodies against cytochrome *c* (1:500), Bcl-2 (1:2000), Bcl-x_L_ (1:2000), Bax (1:2000), Bak (1:2000), PARP (1:500), caspase 3 (1:500), caspase 9 (1:500) or β-actin (1:10,000) for 1 h at room temperature. After undergoing 3 consecutive washes, the blots were incubated with a peroxidase-conjugated donkey anti-rabbit secondary antibody (1:3000 dilution) for 1 h at room temperature. Signals were visualized by enhanced chemiluminescence with Kodak X-OMAT LS film (Eastman Kodak, Rochester, NY, USA) [56].

### 4.12. Caspases Activity Assay

Cells were treated with BL-038 (5 µM) for 24 h, and the enzymatic activity of caspase-3 and caspase-9 in the cell lysate was detected by the Caspase-Glo^®^ 3/7 and Caspase-9 Assay Kits (Promega, Madison, WI, USA), as described in the manufacturer’s protocol. After conducting the treatments according to the experimental design, the homogeneous caspase-3/7 or caspase-9 reagent was added to the 1 × 10^5^ cell sample. Following incubation at room temperature for 2 h, caspase-3/7 and caspase-9 activity, and luminescence was determined using a Veritas microplate luminometer [57].

### 4.13. Quantitative Real-Time PCR (qPCR)

Total RNA was extracted from cells using a TRI-Reagent^®^ (Sigma Aldrich, St. Louis, MO, USA) and 2 µg RNA was used for synthesis of complementary DNA (cDNA) by reverse transcriptase (Invitrogen, Carlsbad, CA, USA). Quantitative real-time PCR was carried out using KAPA SYBR Green (KAPA Biosystem, Woburn, MA, USA) according to the manufacturer’s protocol and reactions were run on the StepOne Plus™ real-time PCR machine (Applied Biosystems, Foster City, CA, USA). The reaction conditions were 10 min at 95 °C for polymerase activation and 40 cycles of 15 s at 95 °C and 60 s at 60 °C. The following primers were used to amplify target genes: human matrix metalloproteinase-9 forward (5′-AGCTGGCAGAGGAATAC-3′), matrix metalloproteinase-9 reverse (5′-CCCCAGAGATTTCGACTC-3′), human VEGF forward (5′-CTACCTCCACCATGCCAAGT-3′), VEGF reverse (5′-GCAGTAGCTGCGCTGATAGA-3′), human GAPDH forward (5′-AGGGCTGCTTTTAACTCTGGT-3′), and GAPDH reverse (5′-CCCCACTTGATTTTGGAGGGA-3′). The expression levels of matrix metalloproteinase-9 or VEGF were determined by normalizing to that of GAPDH. The threshold cycle (*C*_t_) was set above the non-template control background and within the linear phase of amplification of target genes, in order to calculate the cycle numbers at which the transcript was detected (denoted *C*_t_). Each sample was assayed in triplicate and the data shown are representatives of three independent experiments.

### 4.14. Migration Assay

Cells (1.5 × 10^4^) in serum-free medium (200 μL) were added to the upper chamber of a Transwell insert, and vehicle control or BL-038 in serum-free medium (300 μL) was applied to the lower chamber, followed by incubation at 37 °C. After 24 h, cells were fixed and stained with 0.05% crystal violet. Stained cells in the lower chamber were counted.

### 4.15. Statistics

All data are presented as the means ± SEM. Statistical analysis used the Student’s *t*-test or one-way analysis of variance with Bonferroni’s post-hoc test. *p* < 0.05 was considered to be significant.

## 5. Conclusions

We found an anti-tumor effect of BL-038 through the generation of ROS and mitochondrial dysfunction, leading to mitochondrial permeability, cytochrome *c* release, and finally caspase activation. We have demonstrated that BL-038-induced apoptosis in human chondrosarcoma cells is mediated by the caspase-dependent apoptotic pathway. Our research suggests that BL-038 is a potential antitumor drug with multifunctional effects in chondrosarcoma cells.
